# An Untapped and Undocumented Butterfly Diversity in a Rapidly Urbanizing and Fragmenting Forest Habitat in Pokhara, Nepal: First Checklist and Implications for Conservation and Ecotourism

**DOI:** 10.1002/ece3.71937

**Published:** 2025-08-18

**Authors:** Sajan KC

**Affiliations:** ^1^ Institute of Agriculture and Animal Science Tribhuvan University Lamjung Gandaki Province Nepal

**Keywords:** butterfly tourism, conservation, habitat fragmentation, habitat loss, Himalaya

## Abstract

Butterflies serve as sensitive bioindicators of ecological health, with their diversity affected by anthropogenic disturbances such as urbanization and habitat alteration. Lakeside, located in Pokhara, Nepal, is one of the most urbanized areas in the country, with its eastern boundary abutting the Methlang Forest area. Despite its rich biodiversity, the forest remains under‐documented. Between 2017 and 2021, the author conducted modified Pollard Walk surveys on an opportunistic basis to assess the species composition, richness, and seasonal distribution of adult butterflies across the forest. A total of 225 butterfly species, representing six families across 122 genera, were recorded within a 2.1 km^2^ area. Species richness exhibited an annual bimodal pattern, with the highest diversity observed in May (*n* = 107) and April (*n* = 102) followed by October (*n* = 90) and the lowest in December (*n* = 33) and January (*n* = 61). Notable findings included the first record of *Burara anadi anadi* (de Nicéville, 1884) in Nepal, along with sightings of *Pithauria murdava* (Moore, 1866), *Prosotas bhutea* (de Nicéville, [1884]), and *Liphyra brassolis brassolis* (Westwood, 1864), all of which were documented after at least three decades in Nepal. This study represents the first checklist of butterflies in this highly urbanized and ecologically fragmenting tourism hub, underscoring the urgent need for conservation initiatives. The prospects of butterfly tourism, alongside other forms of wildlife tourism, are discussed as a solution to balance habitat conservation with economic development, potentially extending across the broader Himalayan region.

## Graphical abstract

Documenting indicator taxa such as butterflies is crucial in biodiversity hotspots such as the Himalayas, particularly in rapidly urbanizing areas such as Pokhara, Nepal. This study recorded 225 butterfly species in the Methlang Forest area of Lakeside, Pokhara, including new and long‐absent national records. Seasonal richness patterns highlight the need for conservation, with butterfly tourism proposed as a sustainable strategy to balance biodiversity preservation and economic growth.

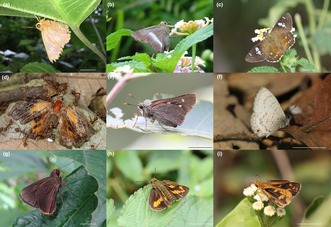

## 1 Introduction

Butterflies are sensitive bioindicators of ecological health owing to their host‐ and habitat‐specificity, and responsiveness to environmental changes (Brown Jr. 1991; Parmesan 1996; Parmesan et al. 1999). This sensitivity is pronounced in areas experiencing urbanization and habitat fragmentation (Clark et al. 2007; Soga and Koike 2012; Kuussaari et al. 2021), such as Lakeside in Pokhara, Nepal, located in the Himalayas—one of the world's biodiversity hotspots (Mittermeier et al. 2004). Colin Smith conducted a comprehensive, multidecade survey of Nepal's butterfly fauna, recording a total of 661 species, including those found in Pokhara, as presented in Smith (2010). Smith (2011a) documented 251 species of butterflies from three locations in the Pokhara Valley. However, rapid urban expansion, fueled by population growth and tourism, has accelerated in Pokhara in recent years, particularly in Lakeside, one of the country's most urbanized areas (Parajuli and Paudel 2014; Pokharel 2020; Khatiwada and Adhikari 2021). Although some studies indicate a slight increase in overall forest cover in the Pokhara Valley in recent years (Rimal et al. 2015; Raut et al. 2020), urban expansion has led to habitat fragmentation, driven by deforestation for hotels and resorts, road construction, and forest loss in areas such as Lakeside (Shrestha and Upadhayaya 2013; Upadhyay 2020; Kunwar et al. 2022). Additionally, this expansion has caused further fragmentation through subsequent soil erosion and landslides (Rimal et al. 2015; Vuillez et al. 2018). Urbanization has been shown to largely reduce butterfly species richness and alter community composition worldwide, primarily through habitat fragmentation, degradation, and the replacement of native vegetation with impervious surfaces (Posa and Sodhi 2006; Clark et al. 2007; Di Mauro et al. 2007; Seto et al. 2012; Fang et al. 2023). These disruptions lead to the loss of larval host plants, diminished nectar sources, altered microclimates, and increased population isolation—factors that collectively diminish both taxonomic and functional diversity, particularly affecting habitat specialists and sensitive species (Clark et al. 2007; Iserhard et al. 2019; Pignataro et al. 2025). This underscores the urgent need for ecological research and targeted conservation in rapidly urbanizing landscapes.

The Methlang Forest area, situated northeast of Lakeside, spans approximately 2.1 km^2^ with an average elevation of 900 m (Google Earth 2024). In recent years, the area has undergone rapid development, primarily through the construction of small to large hotels, spurred by the growth of alternative lodging options such as AirBnBs (Paudel 2021, personal observation as a local resident). As Pokhara's economy relies heavily on tourism (Sharma 2018), the Methlang Forest area is increasingly valued for its panoramic views of Phewa Lake and the Annapurna massif. Despite the encroachment, the forest supports a rich diversity of butterfly species. KC (2020, 2021, 2022, 2023) and KC and Sapkota (2022) documented several notable species from this forest, including *Burara anadi anadi* (de Nicéville, 1884), *Celaenorrhinus pyrrha* (de Nicéville, 1889), and *Aeromachus pygmaeus* (Fabricius, 1775)—species rarely or never recorded elsewhere in Nepal. Additionally, van der Poel (2020) recorded *Eurema andersoni jordani* Corbet and Pendlebury, 1932, from this forest area, and van der Poel (2024) recorded *Coladenia pinsbukana* (Shimonoya and Murayama, 1976) from a nearby location—both representing first records for Nepal. These findings suggest Methlang and its surrounding forests could be a vital biodiversity hotspot, hosting rare and potentially endangered butterfly fauna, as well as other taxa that warrant further study.

The ongoing urbanization of the Methlang Forest area poses a prominent threat to its ecological integrity, emphasizing the urgent need to document its biodiversity and establish baseline data, particularly for indicator taxa such as butterflies. Effective conservation strategies are essential to mitigate the impact of development and preserve the region's ecological richness, a principle that applies to much of the Himalayan region (Sharma et al. 2010; Ali et al. 2024). Pokhara's thriving tourism industry presents a unique opportunity to integrate conservation with economic growth. Ecotourism, which promotes the sustainable use of natural resources, has emerged as a growing trend that fosters both environmental awareness and economic development worldwide (Mpand et al. 2014; Backman and Munanura 2015; Wondirad 2019; Checa et al. 2024), with most studies being conducted in Asia (Wondirad 2019). Charismatic wildlife, such as butterflies, attract global tourists (Whelan 2012; Monterrubio et al. 2013), and while butterfly tourism was previously discontinued in Nepal owing to lack of interest (Smith 2010), the rise of digital marketing and the integration of butterfly tourism with other wildlife activities such as birdwatching and mammal viewing offers potential for revitalization. Additionally, the nearby Rani Forest, located across Phewa Lake and less impacted by urbanization, presents further opportunities for wildlife tourism and conservation (Khatiwada 2013). Documenting and promoting the biodiversity of the Methlang Forest area, particularly its butterfly species, can help Lakeside foster ecotourism and support the long‐term conservation of its natural habitats. While the forest was more pristine in earlier decades, as evidenced by historical imagery available on Google Earth (https://earth.google.com/), the absence of historical butterfly diversity data prevents a definitive conclusion on whether diversity has declined.

This paper provides a checklist of butterfly species recorded over approximately 5 years in the Methlang Forest area by the author, detailing their seasonality and local rarity to establish a baseline for future comparisons. Although records of new seasonal, distributional, or elevational ranges from this study were shared with van der Poel and Smetacek (2022), these data were generalized in their catalog without specifying the study area, highlighting the need for a catalog tailored to this specific study area. This paper also explores the potential for butterfly tourism as a strategy to balance the growing influx of tourists with the conservation of the region's fragile habitats.

## 2 Material and Methods

### 2.1 Study Area

**FIGURE 1:**
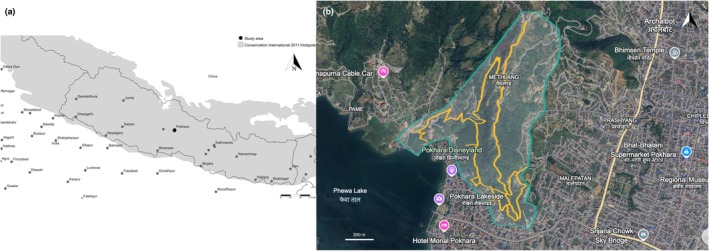
Maps of the study area (a) Map of Nepal highlighting the study area with a black circle, and the Himalayas shaded in gray as a biodiversity hotspot visualized using SimpleMappr (https://www.simplemappr.net) (b) Detailed map of the Methlang Forest area, outlined with a teal border, showing survey trails marked in yellow (~9.2 km) visualized using Google Earth (https://earth.google.com).

Methlang is in the northwestern part of Pokhara, within Kaski District, Nepal, at coordinates 28.227587, 83.963577 (Figure 1). Once part of the Sarangkot Village Development Committee, it is now within Pokhara Metropolitan City. While the name ‘Methlang’ refers to a small village of approximately 0.5 km^2^, the broader forest area—referred to as the Methlang Forest area in this paper (between latitude 28.213056 to 28.235556 and longitude 83.951111 to 83.970833)—covers around 2.1 km^2^ (Google Earth 2024) and is highly vulnerable to urbanization and habitat fragmentation. The area ranges in elevation from 800 m at the foothills to between 1000 and 1100 m at the hilltops, gradually merging with the Sarangkot hill station, which rises to approximately 1700 m above sea level. It is one of the wettest regions in Nepal, receiving an average annual rainfall of 3300 mm (Khadka and Basnet 2019; Shahi et al. 2024). Approximately 80% of the rainfall occurs during the monsoon months (June–September) (Pokharel et al. 2020). The temperature ranges from 5°C to 36°C, with a mean annual temperature of 24°C (DoM 2016; Pathak, Bhuju, et al. 2021; Pathak, Shrestha, et al. 2021), indicating a subtropical climate zone. Seasons are typically classified as spring or premonsoon (March–May), summer or monsoon (June–August), autumn or post‐monsoon (September–November), and winter (December–February) (Mäkelä et al. 2014).

**FIGURE 2:**
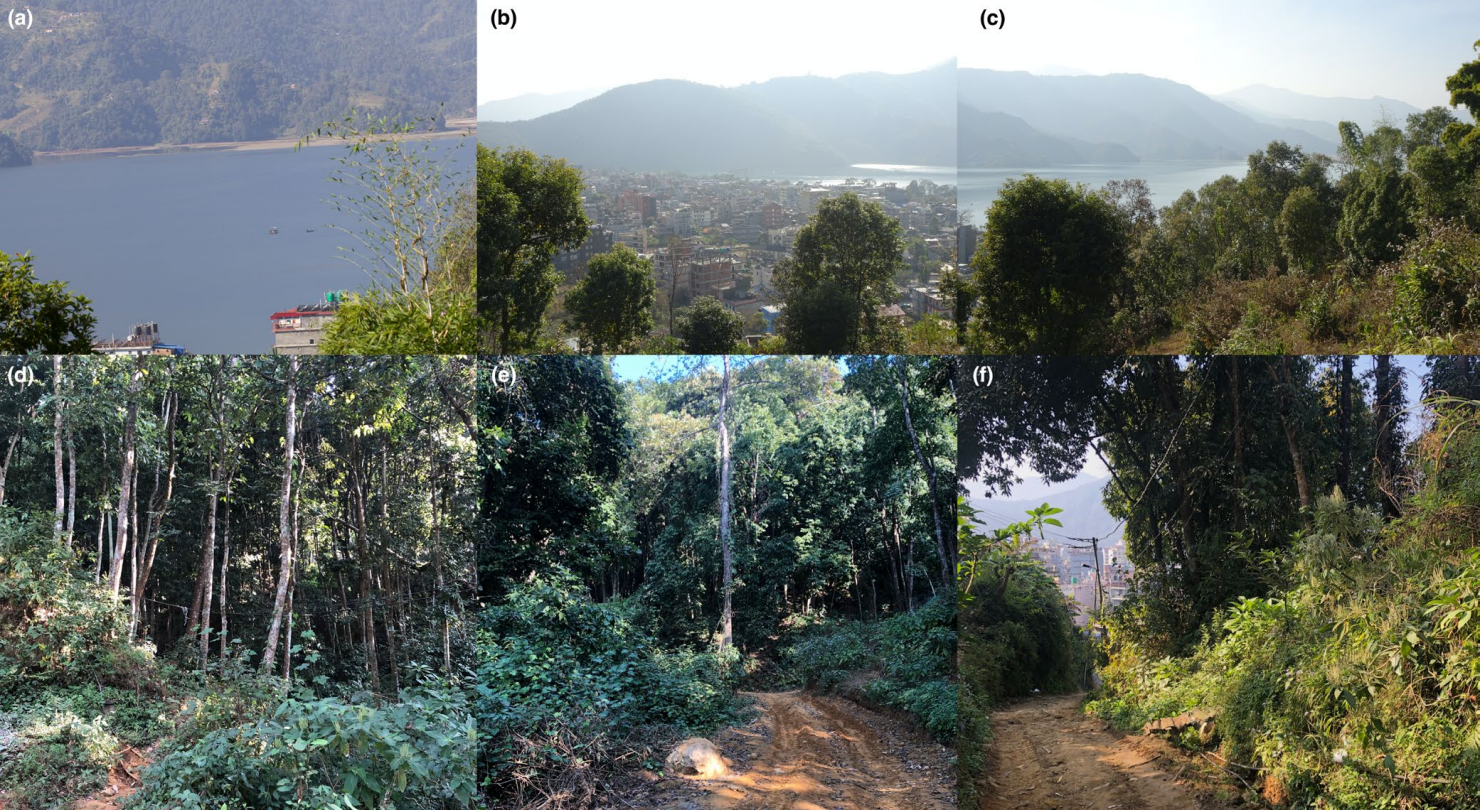
Representative images of habitats within the study area (a) View of Phewa Lake from the study area (b) View of urban Lakeside from the study area (c) Natural vegetation on a hilltop (~900 m) (d) Natural vegetation beside a forest stream in January (e) Survey trail in January (f) Trail leading to the study area in January. Figure 2d–f Rajan KC.

Ecologically, the forest is primarily dominated by the *Schima* Reinw. ex Blume (Theaceae)‐*Castanopsis* (D.Don) Spach (Fagaceae) forest type, typical of the region's mixed deciduous forests (Joshi and Joshi 2009; Basaula et al. 2021). However, native vegetation is increasingly threatened by invasive species such as *Ageratum conyzoides* L. (Asteraceae), *Bidens pilosa* L. (Asteraceae), *Chromolaena odorata* (L.) R.M.King and H.Rob. (Asteraceae), *Eichhornia crassipes* (Mart.) Solms (Pontederiaceae), and *Lantana camara* L. (Verbenaceae) which dominate the area (Pathak, Bhuju, et al. 2021; Pathak, Shrestha, et al. 2021). Many of these invasive plant species also serve as important nectar sources for local butterfly populations, potentially influencing their distribution and seasonal dynamics (Potts et al. 2003, Shackelford et al. 2013, Subedi et al. 2021). However, their ecological dominance can suppress native flora, leading to long‐term reductions in host plant availability and larval habitats; this shift may ultimately result in a net negative impact on butterfly diversity and community stability (Tallamy and Shropshire 2009; Burghardt et al. 2010; Heleno et al. 2010). Although these general vegetation patterns were documented, site‐specific plant species composition was not recorded during surveys, which limits direct correlations between host plant availability and butterfly presence. Climatic conditions during the study period (2017–2021) showed an average annual temperature of 19°C, 65.6% relative humidity, and mean daily precipitation of 5.9 mm (NASA Power 2024). The area features a diverse range of habitats, including forest streams, trails, open hilltops, and the urban fringe (Figure 2).

### 2.2 Data Collection and Analysis

Surveys of adult butterflies in the Methlang Forest area (800–1100 m) were conducted between April 2017 and November 2021, following a modified Pollard Walk approach. These surveys were primarily aimed at monitoring species richness across the entire area through the list‐length method (see Szabo et al. 2010). During each survey, multiple designated trails were visited, with the cumulative distance of all trails totaling approximately 9.2 km, as shown in yellow on the map (Figure 1b). The trails were selected to maximize coverage across the main habitat types present in the study area. Trails were chosen based on accessibility, safety, and their ability to represent the diversity of microhabitats in the fragmented landscape. Surveys took place between 10:00 am and 5:00 pm (NPT), with timing determined by the author's availability due to work and study commitments in Lamjung District. Consequently, surveys were not conducted at regular intervals; a limitation compared to the standard Pollard Walk method (Pollard 1977, 1979). While the study did not survey each month equally every year, each month was surveyed at least eight times over the course of the study period. To accommodate the steep and fragmented terrain of the study area, the original Pollard Walk design (Pollard 1977, 1979) was modified by allowing variation in transect length and by following existing natural trails and ridgelines instead of fixed, evenly spaced paths. Unlike the standard method where observations are restricted to a fixed transect, butterflies were recorded along the entire length of each trail. A consistent walking pace was maintained, and while most observations were within approximately 5 m to each side, the sampling approach prioritized broad spatial coverage across diverse microhabitats. Although abundance data and the standard Pollard Walk protocol were not used, the repeated checklist‐style surveys employed here still offer valuable insights. Presence‐only data from such list‐length methods can support species distribution models (SDMs) and help estimate detection probabilities (Van Strien et al. 2013; Isaac et al. 2014). This approach, while limited in certain analyses, remains effective for tracking biodiversity patterns and informing conservation strategies (Szabo et al. 2010).

Butterflies were recorded through photographic evidence, without capturing or explicitly counting the number of specimens. During the first phase (2017–2018), a Gionee P5W smartphone was used to photograph butterflies. From 2018 to 2020, a Sony DSC‐HX90V camera, and in the final phase (2020–2021), a Canon 7D Mark II with a Canon 100 mm f/2.8 L Macro IS USM lens were used. Metadata, including date, location, and elevation, were recorded for all images by the cameras. Species distribution data were cross‐referenced from Smith (2010, 2011a, 2011b, 2011c), Varshney and Smetacek (2015), and van der Poel and Smetacek (2022), with identification based on Evans (1932, 1949), Corbet and Pendlebury (1956), Eliot (1969), Ek‐Amnuay (2012), Smetacek (2017), Inayoshi (2020–2024), and KC's (unpublished) comprehensive identification keys for skippers of Nepal. When possible, identifications were cross‐verified with regional experts mentioned in the Acknowledgments section. Scientific and common names are based on van der Poel and Smetacek (2022), except for two species: *Eurema brigitta* (Stoll, [1780]) and *Caltoris tulsi* (de Nicéville, [1884]), which follow the updated taxonomic status of Irungbam et al. (2023) and Zhang et al. (2023), respectively. A detailed map of the study area was created using SimpleMappr (https://www.simplemappr.net) and Google Earth (https://earth.google.com). Monthly average temperature, precipitation, and relative humidity data were obtained from the NASA POWER Project (version 2.3.6) (NASA Power 2024). The average values of these climatic variables from 2017 to 2021 across the 12 months were then correlated with the cumulative monthly species richness to assess potential climatic influences on butterfly diversity.

To quantify species abundance and rarity, a species abundance index was adopted from KC and Sapkota (2024a):

vF (Very Frequent): Observed in every survey during its recorded months.

F (Frequent): Regularly observed during its recorded months but not in every survey.

fR (Fairly Rare): Observed 3–10 times during the study.

R (Rare): Observed only twice during the study.

vR (Very Rare): Observed only once during the study.

The Rarity Ratio was calculated using the formula: Rarity Ratio = Rare Species (vR + *R* + fR)/Total Species, providing a measure of species rarity. This metric was chosen because it highlights the proportion of species richness composed of rare versus common species, which is particularly relevant in studies where exact abundance data are unavailable. As the list‐length method was adopted in this study, wherein species presence was determined based on photographic evidence, but individual counts were not systematically recorded except for rarer species, commonly used diversity indices such as the Shannon or Simpson indices, which require precise abundance data, could not be applied reliably.

**FIGURE 3:**
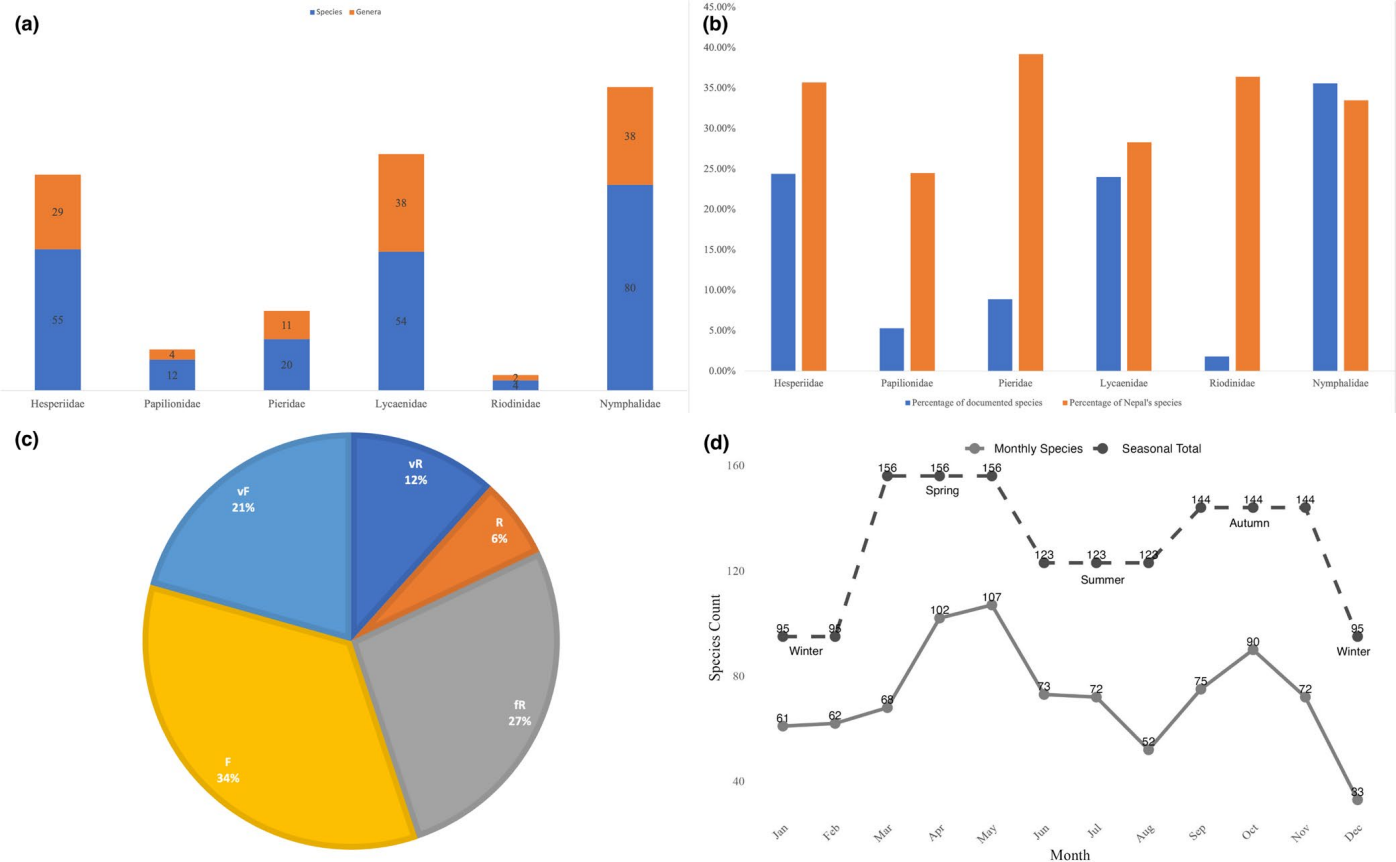
Visual representations illustrating different aspects of species richness (a) Butterfly species (blue) and genera (orange) richness by family observed in the study (b) Percentage comparison of species composition across each family (blue), illustrating the contribution of each family to the total species diversity of Nepal in that family (orange) (c) Percentage distribution of butterfly species by rarity observed in the study. The rarity categories are defined as follows: VF (Very Frequent) species were observed during every survey in their recorded months, F (Frequent) species were regularly observed during their recorded months, fR (Fairly Rare) species were observed 3–10 times throughout the study, R (Rare) species were observed only twice, and vR (Very Rare) species were observed only once (d) Cumulative seasonal variation in species richness within the study area across 5 years, with the *X*‐axis representing months and the *Y*‐axis showing species richness; monthly totals are represented by the solid line and seasonal totals are represented by the dashed line.

**FIGURE 4:**
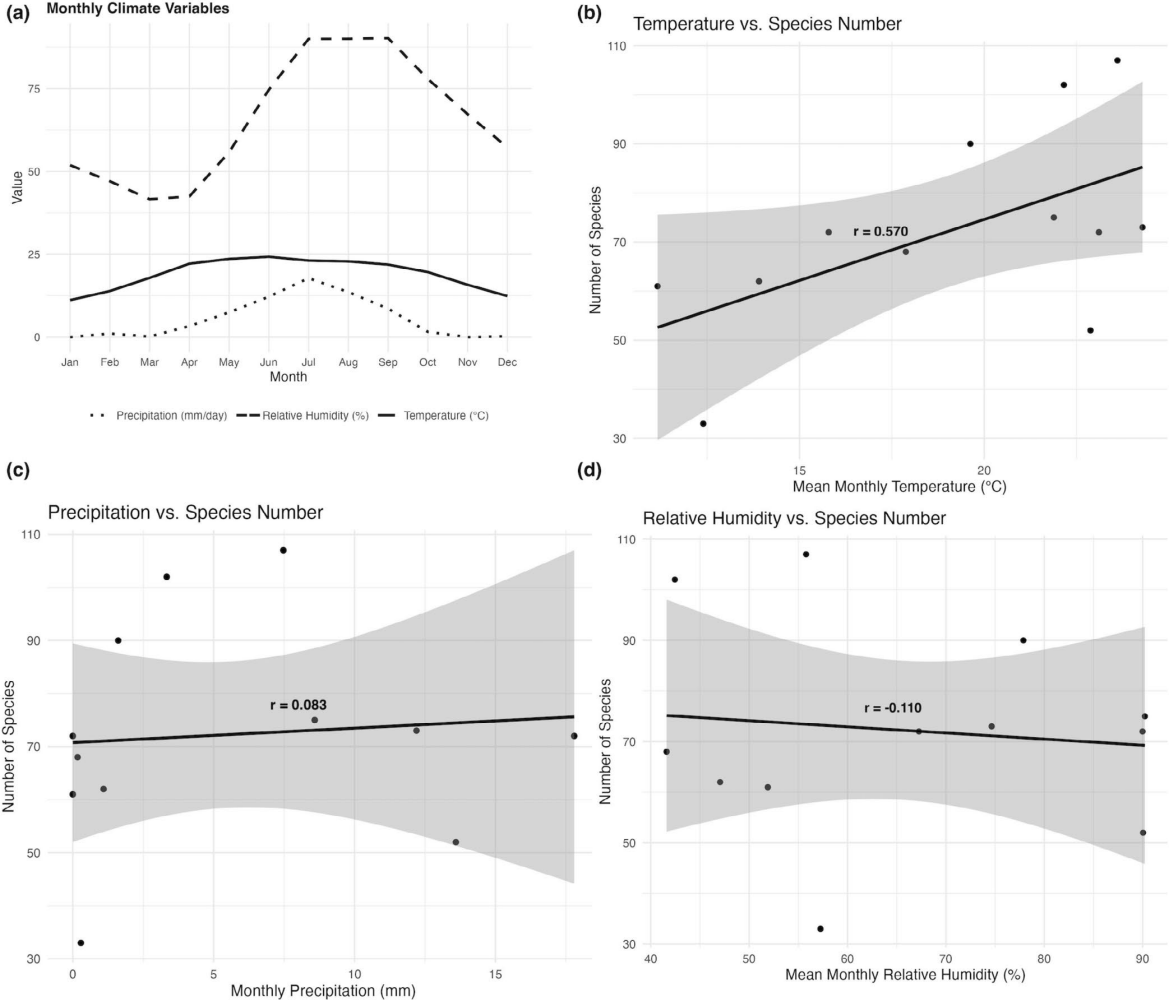
Climatic patterns and their correlation with butterfly species richness across 5 years (2017–2021) (a) Monthly averages of temperature (°C) (middle solid line), precipitation (mm/day) (bottom dotted line), and relative humidity (%) (top dashed line) across 5 years visualized as line graphs, showing seasonal climatic variation across the study period (b) Correlation between cumulative butterfly species richness and average monthly temperature (°C) across 5 years, indicating a moderate positive relationship (*r* = 0.57) (c) Correlation between cumulative species richness and average monthly precipitation (mm/day) across 5 years, showing a very weak positive relationship (*r* = 0.08) (d) Correlation between cumulative species richness and average monthly relative humidity (%) across 5 years, showing a weak negative relationship (r = −0.11).

**FIGURE 5:**
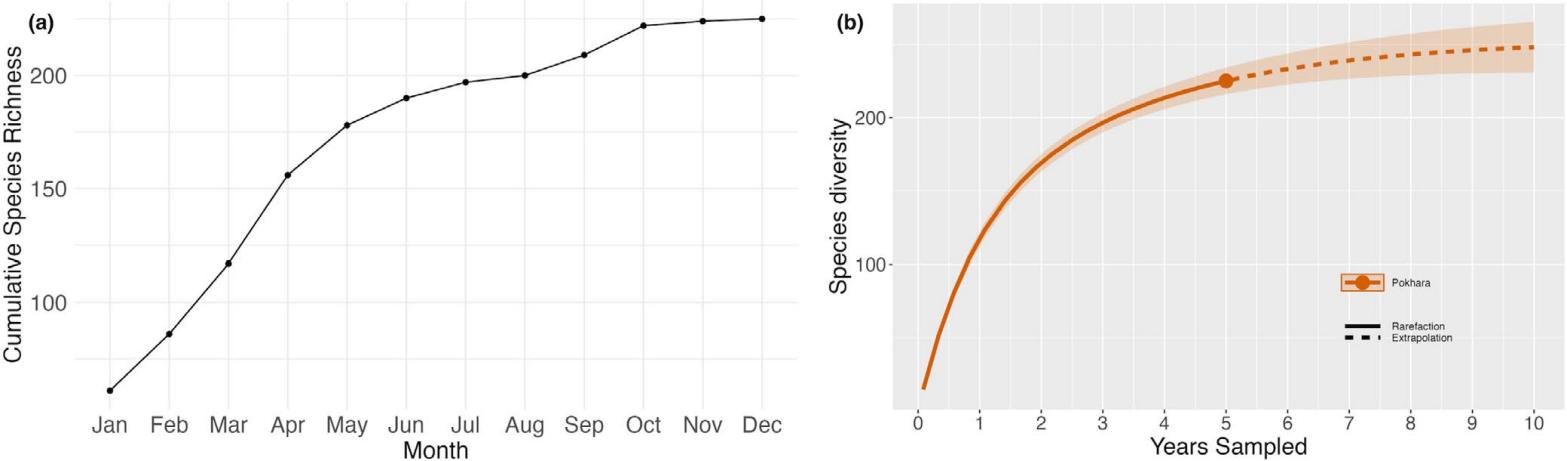
Butterfly species richness patterns based on monthly accumulation and rarefaction analyses over a 5‐year sampling period (2017–2021) (a) Species accumulation curve showing cumulative species richness by month (b) Rarefaction and extrapolation curve based on incidence frequency data across 5 years, showing that observed richness closely approaches the estimated asymptote. Solid and dashed lines represent rarefied and extrapolated richness, respectively, with 95% confidence intervals.

**FIGURE 6:**
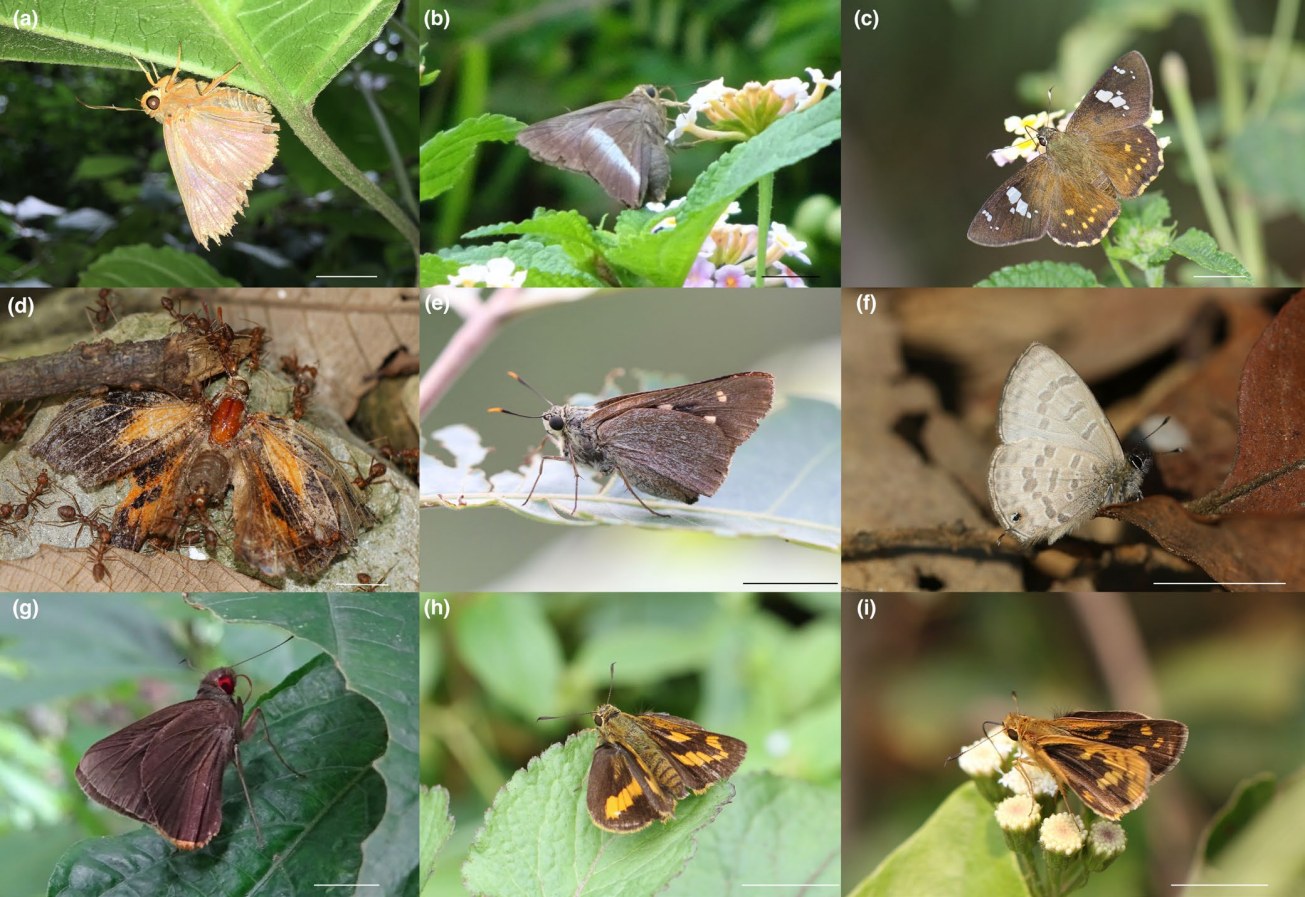
Notable butterfly records from the study area (a) First documented record of *Burara anadi anadi* (de Nicéville, 1884) in Nepal (b) *Hasora taminatus bhavara* Fruhstorfer, 1911 (previously recorded in Nepal only from Lamjung District) (c) *Celaenorrhinus pyrrha* (de Nicéville, 1889) (previously recorded in Nepal only from Dhankuta District) (d) *Liphyra brassolis brassolis* (Westwood, 1864) (seen after 1982 in Nepal) (e) *Pithauria murdava* (Moore, 1866) (seen after 1988 in Nepal) (f) *Prosotas bhutea* (de Nicéville, [1884]) (seen after 1970 in Nepal) (g) *Matapa purpurascens* Elwes and Edwards, 1897 (first few records from Nepal) (h) *Potanthus* cf. *mara mara* (Evans 1932) (if ID correct, second record in Nepal after 1990) (i) *Potanthus* cf. *trachala tytleri* (Evans, 1914) (if ID correct, new province record). (Scale bars = 10 mm).

**FIGURE 7:**
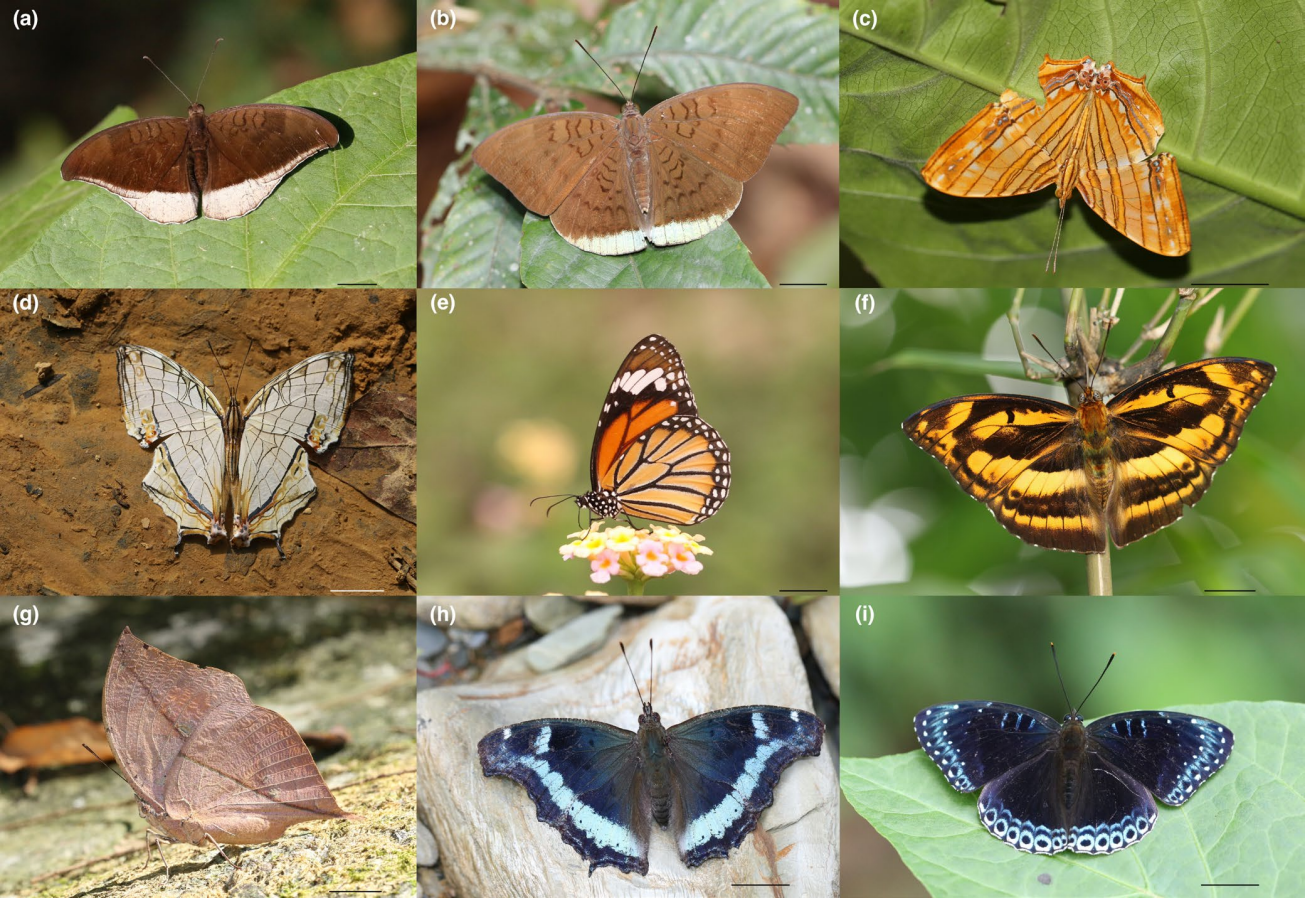
Selected charismatic butterfly species found in the study area (a) *Cynitia lepidea lepidea* (Butler, 1868) (b) *Tanaecia julii appiades* (Ménétriés, 1857) (c) *Chersonesia risa risa* (Doubleday, [1848]) (d) *Cyrestis thyodamas thyodamas* Boisduval, 1846 (e) *Danaus genutia genutia* (Cramer, [1779]) (f) *Abrota ganga ganga* Moore, 1857 (g) *Kallima inachus inachus* (Boisduval, 1846) (h) *Kaniska canace canace* (Linnaeus, 1763) (i) *Stibochiona nicea nicea* (Gray, 1846). (Scale bars = 10 mm).

**FIGURE 8:**
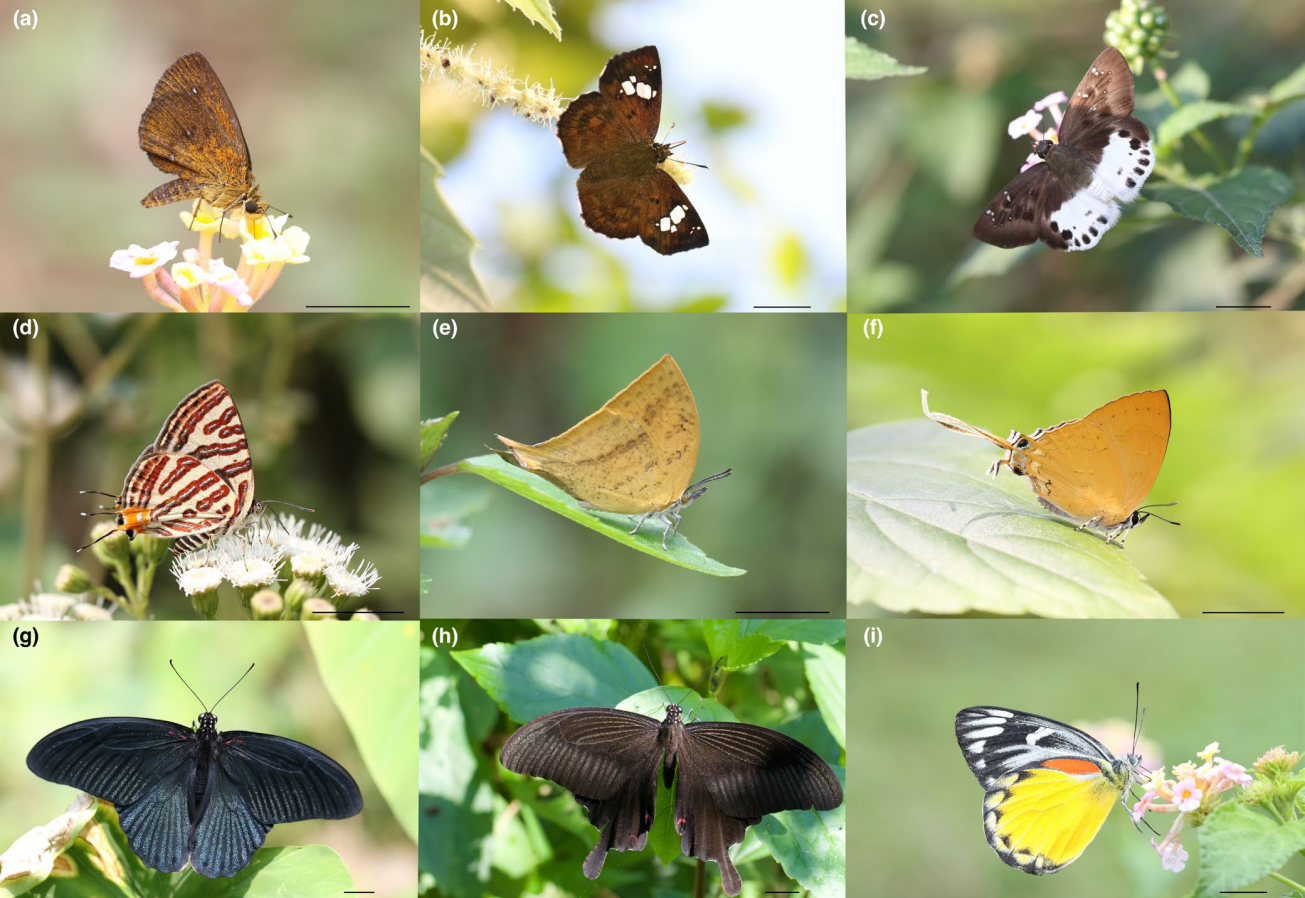
Selected charismatic butterfly species found in the study area (a) *Iambrix salsala salsala* (Moore, [1866]) (b) *Pseudocoladenia fatih fatih* (Kollar, [1844]) (c) *Tagiades menaka menaka* (Moore, [1866]) (d) *Spindasis syama peguanus* Moore, 1884 (e) *Loxura atymnus continentalis* Fruhstorfer, 1912 (f) *Ticherra acte acte* (Moore, [1858]) (g) *Papilio memnon agenor* Linnaeus, 1758 (h) *P*. *helenus helenus* Linnaeus, 1758 (i) *Delias descombesi descombesi* (Boisduval, 1836). (Scale bars = 10 mm).

Seasonal variations in species richness were analyzed to assess temporal changes in butterfly diversity; however, year‐wise analysis was not conducted due to inconsistent sampling across years. Species with unresolved identification or ambiguous records were excluded from abundance classifications to ensure data reliability. For data handling and visualization, MS Excel was used for Figure 3a–c, which present categorical summaries of species richness and composition, including family‐wise richness, contribution to national diversity, and species rarity categories, respectively. For Figure 3d, which illustrates seasonal variation in species richness, RStudio (Version 2023.03.0 + 386) was used. Climatic variables and their correlation with species richness (Figure 4) were analyzed and visualized in RStudio, with Pearson's correlation coefficients calculated using the cor.test() function to evaluate the relationship between species richness and each climatic variable. A species accumulation curve was generated using the specaccum() function in the vegan R package to evaluate sampling completeness over the survey period (Figure 5a). To account for variation in detection and to standardize species richness estimates, rarefaction and extrapolation analysis was performed using the iNEXT package (Chao et al. 2014, 2016) with incidence frequency data, treating each month as a sampling unit (Figure 5b). The analysis was conducted for *q* = 0 (species richness) at a 95% confidence interval, and extrapolation extended to 10 years to estimate potential richness if sampling were continued. Data were visualized using base R and ggplot2. Species image plates (Figures 6, 7, 8) were generated using the Mac Preview app (Version 11.0). Scale bars for species illustrated in Figures 6, 7, 8 are based on Smith (2011c) and Ek‐Amnuay (2012).

## 3 Results

A total of 225 butterfly species, representing six families and 122 genera, were documented in the Methlang Forest area between April 2017 and November 2021. These included 80 species from Nymphalidae, 55 from Hesperiidae, 54 from Lycaenidae, 20 from Pieridae, 12 from Papilionidae, and four from Riodinidae (Figure 3a). This accounts for approximately 32% of Nepal's total butterfly species and about 50% of the species found in the hill region of Nepal based on van der Poel and Smetacek (2022) and Smith (2011b), respectively. Specifically, this accounts for 33.5% of the total Nymphalidae in Nepal, 35.7% of Hesperiidae, 28.3% of Lycaenidae, 39.2% of Pieridae, 24.5% of Papilionidae, and 36.4% of Riodinidae. Figure 3b provides a comparison of the species composition across each family and its relationship to the total species diversity of Nepal.

Of the documented species, 27 were classified as very rare (observed only once), 14 as rare (observed twice), 60 as fairly rare (observed 3–10 times), 78 as frequent (commonly seen during their recorded months), and 46 as very frequent (consistently observed) (Table 1, Figure 3c). The Rarity Ratio showed that approximately 45% of species were rare, with about 55% categorized as common, indicating a fairly balanced distribution of species across the study area. The highest proportion of rare species (vR + *R* + fR) was observed in Pieridae (55% of the family total), followed by Hesperiidae (51%). In contrast, the highest proportion of common species (F + vF) was found in Riodinidae (75% of the family total) and Lycaenidae (59%). The number of species in each rarity category by family is presented in Table 1.

**TABLE 1:** Family‐wise rarity indices for butterfly species recorded in the study area. The rarity categories are defined as follows: VF (Very Frequent) species were observed during every survey in their recorded months, F (Frequent) species were regularly observed during their recorded months, fR (Fairly Rare) species were observed 3–10 times throughout the study, R (Rare) species were observed only twice, and vR (Very Rare) species were observed only once. The total number of species in each family is also provided.

Family	vR	R	fR	F	vF	Total
Hesperiidae	12	5	11	16	11	55
Papilionidae	2	1	2	7	0	12
Pieridae	2	1	8	6	3	20
Lycaenidae	5	2	15	22	10	54
Riodinidae	0	0	1	1	2	4
Nymphalidae	6	5	23	26	20	80
Total	27	14	60	78	46	225

Seasonally, May and April recorded the highest number of species, with 107 and 102 species, respectively, while December had the lowest, followed by January with 33 and 61 species, respectively. Figure 3d illustrates an annual bimodal pattern in species richness, with peaks occurring in spring (April–May), followed by a decline until August. Species richness then rises again, reaching a secondary peak in October, before declining during the winter months to a minimum in December. A total of 156 species were recorded in spring (March–May), 123 in summer (June–August), 144 in autumn (September–November), and 95 in winter (December–February) (Figure 3d).

A correlation analysis between the monthly averages of temperature, relative humidity, and precipitation and the cumulative butterfly species richness across 5 years revealed a moderate positive correlation with temperature (*r* = 0.57) (Figure 4b), indicating that species richness tends to increase during warmer months. In contrast, precipitation exhibited a very weak positive correlation (*r* = 0.08) (Figure 4c), and relative humidity showed a weak negative correlation (r = −0.11) (Figure 4d), suggesting that these two climatic factors have limited influence on overall species richness. The species accumulation curve showed a steep increase in cumulative richness from January through May, indicating that many new species were detected early in the year, likely reflecting a peak in seasonal activity (Figure 5a). After October, the curve began to flatten, suggesting that most species detectable by the sampling method had already been recorded, indicating sampling saturation or seasonal decline. Rarefaction and extrapolation analysis based on 5 years of sampling suggested that the observed richness closely approached the asymptote, with extrapolation estimating a marginal increase beyond the observed richness (Figure 5b), indicating that the sampling effort captured the majority of butterfly species in the study area. Notable species records include *Burara anadi anadi* (Figure 6a), documented in Nepal for the first time (see KC 2020); *Hasora taminatus bhavara* Fruhstorfer, 1911 (Figure 6b), previously recorded in Nepal only from Lamjung District (KC and Sapkota 2022, 2024a); *Celaenorrhinus pyrrha* (Figure 6c), previously known from Dhankuta District alone in Nepal (see KC 2023); *Liphyra brassolis brassolis* (Westwood, 1864) (Figure 6d), rediscovered in Nepal after being unrecorded since 1982 (see KC 2022); *Pithauria murdava* (Moore, 1866) (Figure 6e), observed in Nepal for the first time since 1988, marking a range shift of at least 300 km from eastern Nepal (see KC 2020); *Prosotas bhutea* (de Nicéville, [1884]) (Figure 6f), rediscovered in Nepal after its last record in 1970 (see KC 2021); and *Matapa purpurascens* Elwes and Edwards, 1897 (Figure 6g), which remains an exceptionally rare species, with fewer than five records in Nepal (see van der Poel and Smetacek 2022). Two species of *Potanthus* Scudder, 1872 could not be identified to the species level owing to the absence of collected specimens. These include *Potanthus* cf. *mara mara* (Evans 1932) (Fig. 6h), which, if confirmed, would mark the second record in Nepal since 1990, and *Potanthus* cf. *trachala tytleri* (Evans, 1914) (Fig. 6i), which, if confirmed, would represent a new provincial record. A complete list of the documented species, including scientific names, common names, seasonal occurrences, rarity classifications, and natural histories, is provided in Table 2.

**TABLE 2:** List of species documented in the study, including their scientific names, common names, seasonal occurrences, rarity classifications, and natural histories.

S. No.	Sc. Name	Common Name	Season	Rarity	Natural History
Family: Hesperiidae					
Subfamily: Coeliadinae					
1.	*Bibasis sena sena* (Moore, [1866])	Orange‐tailed Awlet	Apr	R	Crepuscular; nectaring on *Lantana*
2.	*Burara anadi anadi* (de Nicéville, [1884])	Plain Orange Awlet	Sep	vR	Crepuscular; forest trail; under leaf
3.	*Burara jaina jaina* (Moore, [1866])	Orange Awlet	Apr–Jun, Aug	fR	Crepuscular; nectaring on *Lantana*; moist rock; resting under leaves
4.	*Burara oedipodea belesis* (Mabille, 1876)	Branded Orange Awlet	Apr	vR	Crepuscular; nectaring on *Lantana*
5.	*Hasora badra badra* (Moore, [1858])	Common Awl	Apr, May, Jul–Nov	F	Forest trails; under leaves
6.	*Hasora taminatus bhavara* Fruhstorfer, 1911	White‐banded Awl	Sep	vR	Crepuscular; nectaring on *Lantana*
Subfamily: Pyrginae					
7.	*Spialia galba galba* (Fabricius, 1793)	Indian Skipper	Feb–Jun, Aug–Oct	vF	Open areas; nectaring on *Bidens*
Subfamily: Tagiadinae					
8.	*Celaenorrhinus nigricans nigricans* (de Nicéville, 1885)	Small‐banded Flat	Mar–Oct	F	Forest trails; under leaves; on bird droppings
9.	*Celaenorrhinus patula* de Nicéville, 1889	Large Spotted Flat	Mar	vR	Forest trail; nectaring on *Ageratum*
10.	*Celaenorrhinus pyrrha* de Nicéville, 1889	Double‐spotted Flat	May, Nov	fR	Forest streams; under leaves; nectaring on *Lantana* flowers
11.	*Celaenorrhinus dhanada dhanada* (Moore, [1866])	Himalayan Yellow‐banded Flat	Apr, May, Aug–Oct	F	Forest trails; bird droppings
12.	*Celaenorrhinus leucocera* (Kollar, [1844])	Common Spotted Flat	Feb–Nov	vF	Forest trails; gardens; bird droppings
13.	*Coladenia agnioides* Elwes and Edwards, 1897	Elwes' Pied Flat	May, Jun	fR	Forest streams
14.	*Odontoptilum angulata angulata* (C. Felder, 1862)	Chestnut Angle	Apr	vR	Nectaring on *Lantana* flowers
15.	*Pseudocoladenia fatih fatih* (Kollar, [1844])	Himalayan Pied Flat	Mar–Jul, Oct, Nov	vF	Forest trails; forest streams; bird droppings
16.	*Sarangesa dasahara dasahara* (Moore, [1866])	Common Small Flat	Feb–Jun, Sep–Nov	vF	Forest trails; open areas; residential areas; gardens
17.	*Tagiades gana athos* Plötz, 1884	Suffused Snow Flat	Feb, May, Nov	F	Forest trails; bird droppings
18.	*Tagiades litigiosa litigiosa* Fruhstorfer, 1910	Water Snow Flat	Jun–Sep	F	Forest trails; bird droppings; under leaves
19.	*Tagiades menaka menaka* (Moore, [1866])	Spotted Snow Flat	Mar–Jul, Sep, Oct	F	Forest trails
20.	*Tagiades parra gala* Evans 1949	Large Snow Flat	Mar	fR	Forest trails
Subfamily: Hesperiinae					
21.	*Aeromachus dubius impha* Evans, 1943	Dingy Scrub Hopper	Mar, Apr, Jun, Oct	F	Scrubby areas; nectaring on *Ageratum*
22.	*Aeromachus jhora jhora* (de Nicéville, 1885)	Gray Scrub Hopper	Sep	fR	Scrubby areas; nectaring on *Ageratum*
23.	*Aeromachus pygmaeus* (Fabricius, 1775)	Pigmy Scrub Hopper	Apr	vR	Scrubby area; nectaring on *Ageratum*
24.	*Baoris farri farri* (Moore, 1878)	Paintbrush Swift	Mar, Apr, Oct, Nov	F	Forest trails
25.	*Baoris pagana* (de Nicéville, 1887)	Figure of Eight Swift	Oct	vR	Forest trails on bird droppings
26.	*Borbo cinnara* (Wallace, 1866)	Rice Swift	Oct	R	Open areas; near bamboos
27.	*Caltoris cahira austeni* (Moore, [1884])	Colon Swift	Feb–Oct	vF	Forests; forest trails
28.	*Caltoris kumara moorei* (Evans, 1926)	Blank Swift	Apr, Jul–Oct	F	Nectaring on *Lantana* in open areas
29.	*Erionota torus* Evans, 1941	Banana Skipper	Apr, Oct	fR	Forest streams
30.	*Iambrix salsala salsala* (Moore, [1866])	Chestnut Bob	Mar–May, Jul–Nov	vF	Forest trails; streams
31.	*Matapa aria* (Moore, [1866])	Common Redeye	May–Oct	vF	Forest trails; streams; bird droppings
32.	*Matapa druna* (Moore, [1866])	Gray‐brand Redeye	May	R	Forest streams
33.	*Matapa purpurascens* Elwes and Edwards, 1897	Purple Redeye	Sep	vR	Forest stream
34.	*Matapa sasivarna* (Moore, [1866])	Black‐veined Redeye	Oct	R	Forest trails
35.	*Notocrypta curvifascia curvifascia* (C. and R. Felder, 1862)	Restricted Demon	Mar–May, Sep, Oct	vF	Forest trails
36.	*Oriens goloides* (Moore, [1881])	Ceylon Dartlet	Apr, Jun, Jul, Oct, Nov	F	Forest trails; near streams
37.	*Parnara apostata debdasi* Chiba and Eliot, 1991	Sumatran Swift	Apr, Sep	fR	Open areas; nectaring on *Lantana* and other flowers
38.	*Parnara bada bada* (Moore, 1878)	Ceylon Swift	Apr, Jun	F	Open areas; nectaring on *Lantana* and other flowers
39.	*Parnara guttatus mangala* (Moore, [1866])	Straight Swift	Mar–Jun	F	Open areas; nectaring on *Lantana* and other flowers
40.	*Pelopidas agna agna* (Moore, [1866])	Obscure‐branded Swift	Aug–Oct	F	Open areas
41.	*Pelopidas mathias mathias* (Fabricius, 1798)	Small‐branded Swift	Apr–Jun, Aug–Nov	vF	Open areas
42.	*Pelopidas sinensis* (Mabille, 1877)	Large‐branded Swift	Mar, May, Oct	fR	Open areas on wet surfaces
43.	*Pithauria murdava* (Moore, [1866])	Dark Straw Ace	Apr, Sep	R	Forest streams; near *Lantana*
44.	*Potanthus confucius dushta* (Fruhstorfer, 1911)	Chinese Dart	Feb	vR	Open trail by forest
45.	*Potanthus* cf. *mara mara* (Evans 1932)	Sikkim Dart	Oct	vR	Open trail by forest
46.	*Potanthus pallida*	Pale Dart	Oct	vR	Forest trail
47.	*Potanthus pseudomaesa clio* (Evans 1932)	Indian Dart	Mar, Apr, Jul, Sep, Oct	vF	Open areas; nectaring on *Lantana* and other flowers
48.	*Potanthus* cf. *trachala tytleri* (Evans, 1914)	Broad Bi‐dent Dart	Apr	vR	Nectaring on *Lantana* flowers
49.	*Pseudoborbo bevani* (Moore, 1878)	Bevan's Swift	Apr–Oct	vF	Open trails; bird droppings
50.	*Sebastonyma dolopia* (Hewitson, 1868)	Tufted Ace	Apr, May, Sep	fR	Shady areas; nectaring on *Lantana*
51.	*Telicota bambusae* (Moore, 1878)	Dark Palm Dart	Feb–May, Jul–Nov	F	Nectaring on *Lantana* flowers
52.	*Tulsia tulsi tulsi* (Nicéville, 1884) (=*Caltoris tulsi tulsi* (de Nicéville, [1884]))	Purple Swift	Jul, Sep, Oct	fR	Forest trails; on bird droppings; nectaring on *Lantana*
53.	*Zenonoida discreta discreta* (Elwes and Edwards, 1897)	Himalayan Swift	Sep–Nov	F	Forest trails; forest streams
54.	*Zenonoida eltola eltola* (Hewitson, 1869)	Yellow‐spot Swift	Feb, Mar, May, Oct, Nov	F	Forest trails; forest streams
55.	*Zographetus satwa* (de Nicéville, [1884])	Purple and Gold Flitter	Jun–Aug	fR	Open trails; nectaring on *Lantana* flowers
Family: Papilionidae					
Subfamily: Papilioninae					
56.	*Atrophaneura aidoneus* (Doubleday, 1845)	Lesser Batwing	Apr	vR	Open area on *Lantana* flowers
57.	*Graphium doson axionides* (Page and Treadaway, 2014)	Common Jay	Jun, Sep	fR	Forest streams; mud‐puddling
58.	*Graphium sarpedon sirkari* Page and Treadaway, 2013	Common Bluebottle	Feb, May–Aug	F	Open areas on *Lantana* flowers; hilltops; around *Cinnamomum*
59.	*Graphium agamemnon agamemnon* (Linnaeus, 1758)	Tailed Jay	Mar, Jun, Jul	F	Open areas on *Lantana* flowers; hilltops
60.	*Pachliopta aristolochiae aristolochiae* (Fabricius, 1775)	Common Rose	Mar–May	F	Open areas; hilltops
61.	*Papilio alcmenor alcmenor* C. & R. Felder, [1864]	Redbreast	Mar	vR	Nectaring on *Lantana* flowers
62.	*Papilio clytia clytia* Linnaeus, 1758	Common Mime	Jun	fR	Open areas
63.	*Papilio demoleus demoleus* Linnaeus, 1758	Lime Butterfly	Jun, Jul	F	Open areas
64.	*Papilio helenus helenus* Linnaeus, 1758	Red Helen	Mar–Jul, Nov, Dec	F	Open trails
65.	*Papilio memnon agenor* Linnaeus, 1758	Great Mormon	Feb, Mar	F	Open areas; gardens
66.	*Papilio polytes romulus* Cramer, [1775]	Common Mormon	May, Jun, Sep	F	Open areas; gardens; on *Lantana* flowers
67.	*Papilio protenor euprotenor* Fruhstorfer, 1908	Spangle	Sep	R	Near citrus
Family: Pieridae					
Subfamily: Coliadinae					
68.	*Catopsilia pomona pomona* (Fabricius, 1775)	Common Emigrant	Jul	fR	Open trails; nectaring on *Lantana* flowers
69.	*Catopsilia pyranthe pyranthe* (Linnaeus, 1758)	Mottled Emigrant	Feb, May, Jun	fR	Open trails; nectaring on *Lantana* flowers
70.	*Colias fieldii fieldii* Ménétriés, 1855	Dark Clouded Yellow	Mar	fR	Open trails
71.	*Eurema blanda silhetana* (Wallace, 1867)	Three‐spot Grass Yellow	Jan, Feb, Apr, Jul, Nov	F	Forest streams; open trails
72.	*Eurema drona* (Horsfield, 1829) (=*E. brigitta rubella* (Wallace, 1867))	Small Grass Yellow	Jul, Sep	fR	Open trails
73.	*Eurema hecabe hecabe* (Linnaeus, 1758)	Common Grass Yellow	Mar, Oct, Nov	F	Forest streams; open trails
74.	*Eurema laeta sikkima (Moore, [1896])*	Spotless Grass Yellow	Jan, Feb	F	Open trails
75.	*Gandaca harina assamica* Moore, [1906]	Tree Yellow	Jul	vR	Mud‐puddling on a concrete surface
76.	*Gonepteryx nepalensis* Doubleday, 1847	Himalayan Brimstone	Jan, Mar	F	Open trails
Subfamily: Pierinae					
77.	*Appias lyncida eleonora* (Boisduval, 1836)	Chocolate Albatross	Jan	fR	Forest stream
78.	*Cepora nadina phryne* (Fabricius, 1775)	Common Gull	Apr, May	fR	Open areas
79.	*Delias acalis pyramus* (Wallace, 1867)	Redbreast Jezabel	Sep	R	Nectaring on *Lantana* flowers
80.	*Delias descombesi descombesi* (Boisduval, 1836)	Redspot Jezabel	Apr, Jul, Sep, Oct	vF	Hilltops; open areas; nectaring on *Lantana* flowers
81.	*Delias eucharis* (Drury, 1773)	Common Jezabel	Apr, Aug	fR	Open trails; nectaring on *Lantana* flowers
82.	*Delias hyparete* indica (Wallace, 1867)	Painted Jezabel	Apr, May, Aug, Sep	F	Forest streams; open areas; nectaring on *Lantana* flowers
83.	*Delias pasithoe dione* (Drury, [1773])	Redbase Jezabel	Jan, Apr, Aug–Oct	vF	Hilltops; open areas; nectaring on *Lantana* flowers
84.	*Leptosia nina nina* (Fabricius, 1793)	Psyche	Apr	vR	Forest stream
85.	*Pieris brassicae nepalensis* Gray, 1846	Large Cabbage White	Mar–May	F	Open areas; nectaring on *Lantana* flowers
86.	*Pieris canidia indica* Evans, 1926	Indian Cabbage White	Jan–Dec	vF	Open areas; nectaring on *Lantana* flowers
87.	*Pontia daplidice moorei* (Röber, [1907])	Bath White	Mar	fR	Open areas; nectaring on *Lantana* flowers
Family: Lycaenidae					
Subfamily: Aphnaeinae					
88.	*Spindasis lohita himalayanus* (Moore, 1884)	Long‐banded Silverline	May, Sep	fR	Open trails
89.	*Spindasis syama peguanus* Moore, 1884	Club Silverline	Mar, Apr, Sep	F	Open trails
Subfamily: Curetinae					
90.	*Curetis bulis bulis* (Westwood, 1852)	Bright Sunbeam	Apr–Jul	F	Open trails
Subfamily: Lycaeninae					
91.	*Heliophorus epicles latilimbata* Eliot, 1963	Purple Sapphire	Jan–Dec	vF	Open trails
92.	*Heliophorus indicus* (Fruhstorfer, 1908)	Indian Purple Sapphire	Jan–Dec	vF	Open trails
Subfamily: Miletinae					
93.	*Liphyra brassolis brassolis* Westwood, 1864	Moth Butterfly	May	vR	One dead individual on a dry streambed
94.	*Spalgis epius epius* (Westwood, 1852)	Apefly	Nov	vR	Open trail near forest stream
95.	*Taraka hamada mendesia* Fruhstorfer, 1918	Forest Pierrot	Jan, Aug–Oct	F	Open trails; bamboo clumps
Subfamily: Polyommatinae					
96.	*Acytolepis puspa gisca* (Fruhstorfer, 1910)	Common Hedge Blue	Jan–Jul, Nov, Dec	vF	Open trails; forest streams; oviposits on rose buds
97.	*Anthene emolus emolus* (Godart, [1824])	Ciliate Blue	Feb, Jul–Nov	F	Open trails
98.	*Caleta elna noliteia* (Fruhstorfer, 1918)	Elbowed Pierrot	Jul	R	Open trails
99.	*Castalius rosimon rosimon* (Fabricius, 1775)	Common Pierrot	May, Jun	F	Open trails
100.	*Catochrysops strabo strabo* (Fabricius, 1793)	Forget‐me‐not	Jan, Mar–Jun	F	Open trails; forest streams
101.	*Celastrina argiolus iynteana* (de Nicéville, 1884)	Hill Hedge Blue	Jan, Feb	fR	Forest streams
102.	*Celastrina lavendularis limbata* (Moore, 1879)	Plain Hedge Blue	Apr, May	fR	Forest streams
103.	*Celatoxia marginata* (de Nicéville, 1884)	Margined Hedge Blue	Apr	R	Forest stream; nectaring on *Bidens* flowers
104.	*Cupido lacturnus assamica* Tytler, 1915	Indian Cupid	Mar–Sep	vF	Open areas
105.	*Euchrysops cnejus cnejus* (Fabricius, 1798)	Gram Blue	Feb, Jul, Aug, Nov	F	Open areas
106.	*Jamides alecto alocina* Swinhoe, 1915	Metallic Cerulean	Jan, May, Jun, Sep, Nov, Dec	F	Open areas and forest trails
107.	*Jamides bochus bochus* (Stoll, [1782])	Dark Cerulean	Jan, Apr, Jun, Nov, Dec	F	Open areas and forest trails
108.	*Jamides celeno aelianus* (Fabricius, 1793)	Common Cerulean	Jan, Feb, Jul–Nov	vF	Open areas and forest trails
109.	*Lampides boeticus* (Linnaeus, 1767)	Peablue	Jan–Jun	F	Open areas; gardens
110.	*Leptotes plinius plinius* (Fabricius, 1793)	Zebra Blue	Feb–May	fR	Open trails
111.	*Lestranicus transpectus* (Moore, 1879)	White‐banded Hedge Blue	Feb–Apr, Jul, Sep, Oct	F	Forest streams
112.	*Luthrodes pandava pandava* (Horsfield, [1829])	Plains Cupid	Jun, Jul, Sep, Oct	fR	Open areas
113.	*Nacaduba kurava euplea* Fruhstorfer, 1916	Transparent Six‐Lineblue	Jul, Oct	fR	Hilltops; forest trails
114.	*Prosotas bhutea* (de Nicéville, [1884])	Bhutya Lineblue	Jan	fR	Forest streams
115.	*Prosotas nora nora* (C. Felder, 1860)	Common Lineblue	Jan, Apr–Jun, Aug, Oct–Dec	F	Forest streams
116.	*Prosotas pia marginata* Tite, 1963	Margined Lineblue	Jan, Feb, Jul–Dec	vF	Forest streams
117.	*Pseudozizeeria maha maha* (Kollar, [1844])	Pale Grass Blue	Jan–Jun	vF	Open areas
118.	*Udara dilecta dilecta* (Moore, 1879)	Pale Hedge Blue	Jan, Feb, Apr, Jul	F	Forest streams
119.	*Zizeeria karsandra* (Moore, 1865)	Dark Grass Blue	Jan, Apr, May, Sep, Nov, Dec	F	Open areas; vegetable gardens
120.	*Zizina otis indica* (Murray, 1874)	Lesser Grass Blue	Jan–Jun	vF	Open areas; vegetable gardens
121.	*Zizula hylax hylax* (Fabricius, 1775)	Tiny Grass Blue	May, Jun	fR	Open trail around *Lantana* bushes
Subfamily: Theclinae					
122.	*Arhopala centaurus pirithous* (Moore, [1884])	Centaur Oakblue	Jan, May, Aug, Oct	F	Forest streams; hilltops
123.	*Arhopala oenea* (Hewitson, 1869)	Hewitson's Dull Oakblue	Jan, Mar, Apr, Oct, Nov	F	Forest trails
124.	*Arhopala paramuta paramuta* (de Nicéville, [1884])	Hooked Oakblue	Jan–Nov	vF	Forest trails
125.	*Flos areste* (Hewitson, 1862)	Tailless Plushblue	Jan–Mar	F	Forest trails; forest streams
126.	*Flos chinensis* (C. and R. Felder, 1865)	Chinese Plushblue	May, Jul–Sep	fR	Forest trails; forest streams
127.	*Horaga onyx onyx* (Moore, 1858)	Common Onyx	Jul, Sep–Nov	fR	Forest trails; forest streams
128.	*Hypolycaena erylus himavantus* Fruhstorfer, 1912	Common Tit	Oct, Nov	fR	Forest streams
129.	*Iraota timoleon timoleon* (Stoll, [1790])	Silverstreak Blue	Oct, Nov	fR	Forest trails; forest streams
130.	*Loxura atymnus continentalis* Fruhstorfer, 1912	Yamfly	Jan, May, Jul, Sep–Dec	F	Forest trails; forest streams
131.	*Rapala manea schistacea* (Moore, 1879)	Slate Flash	Apr–Jun, Oct, Nov	F	Forest trails; open trails; forest streams
132.	*Rapala nissa nissa* (Kollar, [1844])	Common Flash	Jun	fR	Hilltops; open trails
133.	*Rapala pheretima petosiris* (Hewitson, 1863)	Copper Flash	Oct, Nov	fR	Hilltops; patrolling
134.	*Rapala rectivitta* (Moore, 1879)	Scarce Shot Flash	Jun	vR	Nectaring on *Lantana* flowers
135.	*Rapala scintilla scintilla* de Nicéville, 1890	Scarce Slate Flash	Oct	vR	Hilltops; open trails
136.	*Rapala tara* de Nicéville, [1889]	Assam Flash	Jun	vR	Open areas; patrolling
137.	*Rapala varuna gebenia* Fruhstorfer, 1914	Indigo Flash	May, Oct	fR	Open areas
138.	*Sinthusa chandrana chandrana* (Moore, 1882)	Broad Spark	Jan–Jul	vF	Open trails patrolling along *Lantana* bushes
139.	*Surendra quercetorum quercetorum* (Moore, [1858])	Common Acacia Blue	Jan, Feb, Aug, Nov	F	Forest streams; open trails
140.	*Ticherra acte acte* (Moore, [1858])	Blue Imperial	May, Oct, Nov	F	Forest streams; forest trails
141.	*Zeltus amasa amasa* (Hewitson, 1865)	Fluffy Tit	Jan, Feb, May, Jul, Oct–Dec	F	Forest streams
Family: Riodinidae					
Subfamily: Nemeobiinae					
142.	*Abisara chela chela* de Nicéville, 1886	Spot Judy	Dec	fR	Forest trails
143.	*Abisara fylla* (Westwood, 1851)	Dark Judy	Jan, Feb, Nov, Dec	vF	Forest trails
144.	*Abisara neophron neophronides* Fruhstorfer, 1914	Tailed Judy	Mar–May, Jul, Oct–Dec	F	Forest trails
145.	*Zemeros flegyas indicus* Fruhstorfer, 1898	Punchinello	Mar, May, Jul, Aug, Nov	vF	Open areas
Family: Nymphalidae					
Subfamily: Acraeinae					
146.	*Acraea issoria issoria* (Hübner, [1819])	Yellow Coster	May, Sep	fR	Open areas
147.	*Cethosia biblis tisamena* Fruhstorfer, 1912	Red Lacewing	Jan, Mar, Apr, Oct	F	Forest trails
148.	*Cethosia cyane cyane* (Drury, [1773])	Leopard Lacewing	Feb–Apr, Sep	F	Forest trails
Subfamily: Apaturinae					
149.	*Hestinalis nama nama* (Doubleday, 1844)	Circe	Oct, Nov	fR	Forest streams
150.	*Sephisa chandra chandra* (Moore, [1858])	Eastern Courtier	May, Nov	R	Forest streams
Subfamily: Charaxinae					
151.	*Charaxes bernardus hierax* C. and R. Felder, [1867]	Tawny Rajah	Jun, Oct	fR	Urban fringes on animal excreta; forests on tree saps
Subfamily: Cyrestinae					
152.	*Chersonesia risa risa* (Doubleday, [1848])	Common Maplet	Jan, Apr, Jul, Oct–Dec	vF	Open areas; forest streams; under leaves
153.	*Cyrestis thyodamas thyodamas* Boisduval, 1846	Common Map	Jan–Apr, Jun, Aug, Nov, Dec	vF	Forest trails; forest streams
Subfamily: Danainae					
154.	*Danaus chrysippus chrysippus* (Linnaeus, 1758)	Plain Tiger	Apr, May, Jul, Nov, Dec	vF	Open areas; nectaring on *Lantana* flowers
155.	*Danaus genutia genutia* (Cramer, [1779])	Common Tiger	Feb–Jun, Oct	vF	Open areas; nectaring on *Lantana* flowers
156.	*Euploea core core* (Cramer, [1780])	Common Indian Crow	Feb–Sep	vF	Open areas; nectaring on *Lantana* flowers
157.	*Euploea klugii kollari* C. and R. Felder	King Crow	Apr, Sep	fR	Open areas; nectaring on *Lantana* flowers
158.	*Euploea mulciber mulciber* (Cramer, [1777])	Striped Blue Crow	Apr–Jul, Sep	F	Open areas; nectaring on *Lantana* flowers
159	*Parantica aglea melanoides* Moore, 1883	Glassy Tiger	Jan–Aug	vF	Open areas; nectaring on *Lantana* flowers
160.	*Tirumala limniace exoticus* (Gmelin, 1790)	Blue Tiger	Apr, May	fR	Open areas; nectaring on *Lantana* flowers
161.	*Tirumala septentrionis septentrionis* (Butler, 1874)	Dark Blue Tiger	Apr, Jul–Sep	F	Open areas; nectaring on *Lantana* flowers
Subfamily: Heliconiinae					
162.	*Phalanta phalantha phalantha* (Drury, [1773])	Common Leopard	Jun, Jul	fR	Open areas
163.	*Vagrans egista sinha* (Kollar, [1844])	Vagrant	Aug–Oct	fR	Forest streams
Subfamily: Limenitidinae					
164.	*Abrota ganga ganga* Moore, 1857	Sergeant‐major	May, Jun, Sep	F	Forest trails; forest streams
165.	*Athyma cama cama* Moore, [1858]	Orange Staff Sergeant	Apr	vR	Nectaring on *Lantana* flowers
166.	*Athyma nefte inara* (Westwood, 1850)	Color Sergeant	Sep	vR	Forest stream
167.	*Athyma perius perius* (Linnaeus, 1758)	Common Sergeant	Aug, Oct, Dec	fR	Forest stream; Nectaring on *Lantana* flowers
168.	*Athyma ranga ranga* Moore, [1858]	Blackvein Sergeant	Jan, Jun, Nov	fR	Forest trails; forest streams
169.	*Athyma selenophora selenophora* (Kollar, [1844])	Staff Sergeant	Jan, Sep–Nov	fR	Forest stream; Nectaring on *Lantana* flowers
170.	*Cynitia lepidea lepidea* (Butler, 1868)	Gray Count	Jan, Apr, May, Jul–Dec	vF	Forest trails; forest streams
171.	*Euthalia aconthea suddhodana* Fruhstorfer, 1913	Common Baron	May, Jun, Oct– Dec	F	Forest trails; forest streams
172.	*Euthalia lubentina lubentina* (Cramer, [1777])	Gaudy Baron	Feb	R	Forest streams
173.	*Moduza procris procris* (Cramer, [1777])	Commander	Feb	R	Moist surfaces near residential areas; forest streams
174.	*Neptis ananta ochracea* Evans, 1924	Yellow Sailer	May, Nov	fR	Forest streams
175.	*Neptis* cf. *capnodes pandoces* Eliot 1969	Eliot's Sailer	Apr, May	fR	Forest streams
176.	*Neptis cartica cartica* Moore, 1872	Plain Sailer	Apr, May	fR	Forest streams
177.	*Neptis clinia susruta* Moore, 1872	Clinia Sailer	Jan, Feb, Apr, May, Jul, Aug, Oct, Nov	F	Forest trails; forest streams
178.	*Neptis hylas kamarupa* Moore, [1875]	Common Sailer	Jan–May, Nov, Dec	vF	Open trails; forest streams
179.	*Neptis nata adipala* Moore, 1872	Nata Sailer	Mar–May, Aug, Sep	F	Forest trails; forest streams
180.	*Neptis radha radha* Moore, 1857	Great Yellow Sailer	Oct	vR	Forest trail
181.	*Neptis sankara amba* Moore, 1858	Broad‐banded Sailer	May	R	Forest trails; forest streams
182.	*Neptis sappho astola* Moore, 1872	Pallas' Sailer	Jan–May, Jul, Sep	vF	Open trails; forest streams
183.	*Neptis soma butleri* Eliot 1969	Creamy Sailer	Jan, Feb, May, Oct	F	Forest trails; forest streams
184.	*Neptis zaida bhutanica* Tytler, 1926	Pale Green Sailer	May	vR	Forest trail near a forest stream
185.	*Pantoporia hordonia hordonia* (Stoll, [1790])	Common Lascar	Sep, Nov	F	Forest trails; forest streams
186.	*Pantoporia sandaka davidsoni* Eliot 1969	Extra Lascar	Feb, Apr	fR	Forest trails; forest streams
187.	*Phaedyma columella ophiana* (Moore, 1872)	Short‐banded Sailer	Jan, May, Aug, Oct, Nov	F	Forest trails; forest streams
188.	*Tanaecia julii appiades* (Ménétriés, 1857)	Common Earl	Jan, Feb, May, Sep–Dec	vF	Forest trails; forest streams
Subfamily: Nymphalinae					
189.	*Aglais caschmirensis aesis* Fruhstorfer, 1912	Indian Tortoiseshell	Jan–May	F	Open areas; open trails; hilltops
190.	*Doleschallia bisaltide indica* Moore, 1899	Autumn Leaf	Nov	R	Forest stream
191.	*Hypolimnas bolina jacintha* (Drury, 1773)	Great Eggfly	Jan, Jul	F	Forest trails near hilltops
192.	*Junonia almana almana* (Linnaeus, 1758)	Peacock Pansy	Jan, Feb, Jun, Jul, Sep	vF	Open areas; open trails
193.	*Junonia atlites atlites* (Linnaeus, 1763)	Gray Pansy	Jan, Jun	fR	Forest streams
194.	*Junonia hierta hierta* (Fabricius, 1798)	Yellow Pansy	Jun	vR	Dry riverbed
195.	*Junonia iphita iphita* (Cramer, [1779])	Chocolate Pansy	Jan, Feb, May, Jun, Aug, Nov	vF	Open areas; open trails
196.	*Junonia lemonias persicaria* (Fruhstorfer, 1912)	Lemon Pansy	Jan–Jul	vF	Open areas; open trails
197.	*Kallima inachus inachus* (Boisduval, 1846)	Orange Oakleaf	Mar–Oct	vF	Forest trails; forest streams
198.	*Kaniska canace canace* (Linnaeus, 1763)	Blue Admiral	Jan, Jun, Sep–Dec	F	Forest trails; forest streams
199.	*Symbrenthia brabira brabira* Moore, 1872	Himalayan Jester	Jan, May	fR	Forest trails; forest streams
200.	*Symbrenthia hypselis cotanda* Moore, [1875]	Spotted Jester	Jan, Apr, May, Oct	F	Forest trails; forest streams
201.	*Symbrenthia lilaea khasiana* Moore, [1875]	Common Jester	Jan–Mar, May, Nov, Dec	vF	Open trails; forest streams
202.	*Vanessa cardui* (Linnaeus, 1758)	Painted Lady	Mar–Jul	vF	Open areas; open trails; hilltops; gardens
203.	*Vanessa indica indica* (Herbst, 1794)	Indian Red Admiral	Jan–Mar, Dec	F	Open areas; open trails; hilltops; gardens
Subfamily: Pseudergolinae					
204.	*Stibochiona nicea nicea* (Gray, 1846)	Popinjay	Apr, May, Aug, Oct–Dec	F	Forest streams
Subfamily: Satyrinae					
205.	*Elymnias hypermnestra undularis* (Drury, 1773)	Common Palmfly	Feb, Apr, Oct–Dec	F	Forest streams
206.	*Elymnias malelas malelas* (Hewitson, 1863)	Spotted Palmfly	Mar–May	fR	Forest streams
207.	*Heteropsis malsara* (Moore, 1857)	White‐line Bushbrown	Mar, May–Aug, Oct	F	Forest floor; forest trails
208.	*Lethe chandica chandica* (Moore, [1858])	Angled Red Forester	Mar–May	F	Forest floor; forest trails
209.	*Lethe confusa confusa* Aurivillius, 1898	Banded Treebrown	Mar, May–Jul, Sep–Dec	vF	Forest floor; forest trails; rotten organic matter
210.	*Lethe europa niladana* Fruhstorfer, 1911	Bamboo Treebrown	Oct, Nov	fR	Near bamboos
211.	*Lethe kansa kansa* (Moore, 1857)	Bamboo Forester	Apr, Nov	fR	Forest floor; forest trails
212.	*Lethe mekara mekara* (Moore, [1858])	Common Red Forester	May, Jul, Oct	fR	Forest floor; forest trails
213.	*Lethe verma sintica* Fruhstorfer, 1911	Straight‐banded Treebrown	May, Nov, Dec	fR	Forest floor; forest trails
214.	*Melanitis leda leda* (Linnaeus, 1758)	Common Evening Brown	Feb, May, Jun, Oct, Nov	F	Forest floor; forest trails
215.	*Melanitis phedima bela* Moore, 1857	Dark Evening Brown	Apr, May, Jul, Oct–Dec	F	Forest floor; forest trails
216.	*Mycalesis adamsoni adamsoni* Watson, 1897	Watson's Bushbrown	Feb–Apr, Jul–Oct	vF	Forest floor; forest trails
217.	*Mycalesis mineus mineus* (Linnaeus, 1758)	Dark‐brand Bushbrown	Jan, Feb, May, Aug	F	Forest floor; forest trails
218.	*Mycalesis perseus blasius* (Fabricius, 1798)	Common Bushbrown	Jan, Feb	fR	Forest stream
219.	*Mycalesis visala visala* Moore, [1858]	Long‐brand Bushbrown	Feb, Jun, Jul, Oct, Dec	F	Forest floor; forest trails
220.	*Orsotriaena medus medus* (Fabricius, 1775)	Jungle Brown	Jan, Feb, May	F	Forest floor; forest trails
221.	*Ypthima avanta* Moore, [1875]	Jewel Fivering	May–Jul	fR	Open areas; open trails
222.	*Ypthima baldus baldus* (Fabricius, 1775)	Common Fivering	Jan–Dec	vF	Open areas; open trails
223.	*Ypthima huebneri* Kirby, 1871	Common Fourring	Jan–Apr, Jul	vF	Open areas; open trails
224.	*Ypthima nareda* (Kollar, [1844])	Large Threering	Oct	vR	Open areas; open trails
225.	*Ypthima newara newara* Moore, [1875]	Newar Threering	Apr–Jun, Sep, Oct	F	Open areas; open trails

## 4 Discussion

### 4.1 Species Richness and Seasonal Variations

The study most comparable to this one is by KC and Sapkota (2024a), who documented 226 butterfly species (cf. 225 in this study) within a 3 km^2^ area (cf. 2.1 km^2^ in this study) in Bhorletar, Lamjung District, located approximately 28 aerial km away from the current study site, over an 18‐month period. Both studies, with species richness exceeding 200, highlight significant biodiversity, likely owing to key ecological factors; both study areas are in ecotonal zones, where the elevation gradient supports a unique mix of species from both higher and lower elevations (McCain and Grytnes 2010; Kark 2013). This ecological diversity likely contributes to the high species count. Additionally, the presence of diverse microhabitats, such as riparian zones, forest edges, and open clearings, provides a range of ecological niches within a small area, further supporting the remarkable species richness observed. The author's taxonomic knowledge and ability to document cryptic or overlooked species has also potentially played a role in the remarkable diversity observed. Examples include complex taxa such as *Potanthus* spp., *Prosotas* Druce, 1891 spp., *Rapala* Moore, [1881] spp., *Parnara* Moore, [1881] spp., *Caltoris* Swinhoe, 1893 spp., and *Celaenorrhinus* Hübner, [1819] spp. Furthermore, the author's potential bias toward selecting suitable habitats, such as riparian areas and nectar sources, for species observation during surveys likely contributed to the high number of recorded observations.

In contrast, studies from neighboring areas with similar elevations and habitats but larger geographical coverage have reported lower species richness. For example, Miya et al. (2021) recorded 149 species in 35 km^2^ in Byas, Lamjung District across nine months; Neupane and Miya (2021) observed 180 species across 146 km^2^ in Putalibazar, Syangja District across 14 months; and Subedi et al. (2021) documented 138 species in 30 km^2^ in Rupa Wetland, Kaski District across 12 months. These studies employed fixed transect line methods, typically along predetermined paths within accessible or representative habitats. Such designs, while consistent and repeatable, often limit the spatial heterogeneity captured during surveys, potentially underrepresenting cryptic, canopy‐dwelling, or microhabitat‐specific species (Pellet et al. 2012; Riva et al. 2020). In contrast, the present study employed a modified Pollard Walk, which incorporated flexible routes across fragmented forest patches, enabling broader habitat coverage and increased chances of detecting diverse species assemblages. Additionally, the longer duration of this study (5 years) improved the likelihood of encountering temporally restricted or rare species. Thus, methodological differences—particularly in spatial coverage and temporal extent—likely contributed to the comparatively higher species richness observed here. Moreover, species richness often stabilizes once a certain area threshold is reached (Colwell and Coddington 1994; Gotelli and Colwell 2001; Chiu 2023). Had this study covered a larger area, such as 40 km^2^, the species count would likely have plateaued at around 250–260 species, as also evident from the extrapolation analysis in time (Figure 5b), suggesting that, although the current study reflects high biodiversity potential, further sampling may yield diminishing returns.

Species richness in the Methlang Forest area follows an annual bimodal pattern, consistent with findings from neighboring (Miya et al. 2021; Neupane and Miya 2021; KC and Sapkota 2024a) and other regions around the world (Gupta et al. 2019; Naik et al. 2022; Sapkota et al. 2024). The species richness peaks in spring, declines until late monsoon or early fall when it peaks again, and then decreases through the winter months (Dec–Feb). Although the irregular survey schedule—dictated by the author's availability—may have introduced some temporal sampling bias, the bimodal pattern of butterfly activity was consistently observed across years and in neighboring areas as well (Miya et al. 2021, Neupane and Miya 2021, KC and Sapkota 2024a), indicating that this is a recurring seasonal trend in this area rather than an artifact of uneven sampling. As discussed by KC and Sapkota (2024a), this bimodal pattern is likely driven by the emergence of new larval host plants in spring, coupled with favorable abiotic conditions and a low initial population of natural enemies such as parasitoids early in the season (see Aiello 1992). The decline in species richness after May may be attributed to high precipitation, elevated temperatures, increased populations of natural enemies, and challenges in conducting surveys during this period. Even when butterflies are present, extreme heat may cause them to remain hidden or exhibit reduced activity levels (Clench 1966). When conditions improve in late monsoon or fall, species richness rebounds.

The moderate positive correlation between temperature and species richness suggests that butterfly activity and detectability in the Methlang Forest are influenced by seasonal temperature increases—a pattern consistent with findings from other tropical and subtropical regions (Gupta et al. 2019; Lazarina et al. 2023; KC and Sapkota 2024a), given that butterflies are ectothermic organisms. In contrast, the weak correlations with precipitation and relative humidity imply that these factors may exert a less direct influence on overall species diversity, though they could still affect the phenology or detectability of specific taxa.

As shown in Table 1, many multivoltine species, such as *Melanitis leda leda* (Linnaeus, 1758), *Mycalesis mineus mineus* (Linnaeus, 1758), *Mycalesis visala visala* Moore [1858], *Eurema blanda silhetana* (Wallace, 1867), *Eurema hecabe hecabe* (Linnaeus, 1758), and *Danaus genutia genutia* (Cramer, [1779]), were recorded intermittently across months. This intermittent recording is likely owing to their low population densities in certain months, rather than their complete absence, or because they may have been present, but no photographic evidence was available to confirm their presence. In addition, species that are more frequently in flight than stationary, such as papilionids, may have been underrepresented. In contrast, many of these species were consistently recorded year‐round by KC and Sapkota (2024a) in Bhorletar, Lamjung District, a more pristine natural habitat for butterflies, which hosted similar species richness but in much higher abundance, highlighting that the current study area may have supported similar diversity in earlier decades. Furthermore, the surveys conducted in Bhorletar were more evenly distributed throughout the months; those surveys relied not only on photographic evidence but also on visual recollections, which were noted after each survey, likely contributing to the consistent records. Although this study does not explicitly detail the abundance of each species, counts of over 400 individuals per day during nonwinter months were common—approximately half the number recorded in Bhorletar, Lamjung. The latter region also had more mud‐puddling areas along two perennial rivers, which often hosted multiple butterflies at a single spot, while the current study area consisted solely of seasonal forest streams, where group mud‐puddling was limited. While the study did not include quantitative spatial mapping of species occurrences across different habitat types, qualitative observations suggest that areas with relatively intact forest patches and riparian zones consistently supported higher species richness compared to open grasslands or heavily disturbed trailsides. These microhabitats likely offer greater structural complexity and host plant diversity, which are important for supporting a range of butterfly species (Koh and Sodhi 2004; Majumder et al. 2012; Miya et al. 2021). Pieridae exhibited the highest percentage of rare species followed closely by Hesperiidae, suggesting that a significant portion of their diversity in the surveyed area comprises species with limited abundance or restricted occurrence. These families may require targeted monitoring to assess population trends and habitat specificity. In contrast, Riodinidae displayed the highest proportion of common species, though this result should be interpreted cautiously due to the low overall sample size for the family, i.e., only four species of which three were common. Lycaenidae also showed a high percentage of common species, possibly reflecting their broader ecological tolerance or higher detectability, particularly on flowers and open trails.

The current checklist is incomplete, as species not recorded in this study have been documented in the study area mostly before the study period. These species include *Acraea terpsicore* (Linnaeus, 1758), *Arhopala eumolphus eumolphus* (Cramer, [1780]), *Arhopala khamti* Doherty, 1891, *Athyma jina jina* Moore, [1858], *Auzakia danava danava* (Moore, [1858]), *Cepora nadina nadina* (Lucas, 1852), *Chliaria kina kina* (Hewitson, 1869), *Cupido huegelii dipora* (Moore, 1865), *Deudorix epijarbas ancus* Fruhstorfer, 1912, *Dodona durga durga* (Kollar, [1844]), *Hypolimnas misippus* (Linnaeus, 1764), *Ixias pyrene latifasciata* Butler, 1871, *Miletus chinensis assamensis* (Doherty, 1891), *Lasippa viraja viraja* (Moore, 1872), *Parantica sita sita* (Kollar, [1844]), *Prosotas dubiosa indica* (Evans, [1925]), *Symbrenthia brabira brabira* Moore, 1872, *Telicota colon colon* (Fabricius, 1775), *Udara albocaerulea albocaerulea* (Moore, 1879), and *Ypthima nikaea* Moore, [1875] along with a few unidentified species (pers. comm., van der Poel, P., 2025). Some of these species were also recorded by the author in areas just outside the forest area, along with several other species observed in the Pokhara Valley but not documented in the current study. This indicates that the species richness in the area extends beyond the number currently reported.

### 4.2 Implications, Threats, and Changes

Butterfly diversity in the Methlang Forest area provides a valuable model for similar habitats in the broader Pokhara region at comparable elevations. Two decades ago, the forest was more contiguous and less fragmented; however, insufficient historical butterfly records make it challenging to draw definitive conclusions about changes in species diversity. A total of 225 species recorded in this study, along with their rarity indices, provide an important baseline for future ecological comparisons.

Without proper management strategies to guide sustainable tourism and urbanization, habitat fragmentation in the region is likely to worsen, impacting not only insect populations but also other wildlife, including bird species in Phewa Lake (see Basaula et al. 2021) and endemic insects such as *Microgomphus phewataali* Conniff and Limbu, 2018 (Conniff and Limbu 2018). Although the local community has likely historically managed forest resources sustainably, recent deforestation driven by tourism development, combined with rising pollution levels, has resulted in substantial habitat loss. Despite community forest management initiatives, the expanding tourism industry poses future threats to the integrity of these ecosystems.

Another significant threat to biodiversity in the region is global climate change. Winter seasons at higher elevations in Nepal are becoming shorter and warmer (Kattel and Yao 2013; Karki et al. 2019; Dhital et al. 2023). Over nearly 5 years of butterfly observations in this study, notable changes were documented. For instance, in 2021, the winter was unusually brief, with over 30 butterfly species, including species such as *Taraka hamada mendesia* (Fruhstorfer, 1918), observed in flight on a single day in mid‐January. The global rise in temperatures may cause the northward expansion of lowland species, while native species in the region could become rarer or shift their distributions (Parmesan and Yohe 2003). A similar trend has been noted in Godavari, Lalitpur District (1500 m), where lowland species are appearing more frequently (pers. comm., Limbu, M., 2024), and this pattern may extend across the Himalayas. Shorter winters can also cause phenological mismatches, potentially reducing the populations of certain species over time (Fabina et al. 2010). Additionally, this study shows that certain species are strongly tied to specific habitats, such as open areas, forest trails, forest floors, and streams (see Table 1). Therefore, habitat alteration through deforestation and clearing is likely to favor species adapted to disturbed areas or synanthropic species such as *Pieris canidia* Linnaeus, 1768, and *P. brassicae* Linnaeus, 1768, potentially shifting species composition and abundance in these ecosystems.

### 4.3 Prospects of Butterfly Tourism

The year‐round butterfly diversity in the Methlang Forest area, with its stunning vistas of Phewa Lake to the west and the Annapurna massif to the north, presents a prime opportunity for butterfly tourism. The region hosts a diversity of large nymphalid species, including *Neptis* Fabricius, 1807 spp., *Cynitia lepidea lepidea* (Butler, 1868) (Figure 7a), *Tanaecia julii appiades* (Ménétriés, 1857) (Figure 7b), *Chersonesia risa risa* (Doubleday, [1848]) (Figure 7c), *Cyrestis thyodamas thyodamas* Boisduval, 1846 (Figure 7d), and *Danaus genutia genutia* (Figure 7e). These species are abundant across multiple seasons, thriving along forest trails, riparian zones, and in flower‐rich open areas. These habitats also offer an ideal setting for butterfly watching and nature photography.

Riparian zones, particularly along forest streams, host species such as *Abrota ganga ganga* Moore, 1857 (Figure 7f), *Athyma ranga ranga* Moore, [1858], *Kallima inachus inachus* (Boisduval, 1846) (Figure 7g), *Kaniska canace canace* (Linnaeus, 1763) (Figure 7h), and *Stibochiona nicea nicea* (Gray, 1846) (Figure 7i) making the area highly accessible for butterfly watchers, even near downtown Lakeside. Smaller species from the families Hesperiidae and Lycaenidae, such as *Iambrix salsala salsala* (Moore, [1866]) (Figure 8a), *Pseudocoladenia fatih fatih* (Kollar, [1844]) (Figure 8b), *Tagiades menaka menaka* (Moore, [1866]) (Figure 8c), *Spindasis syama peguanus* Moore, 1884 (Figure 8d), *Loxura atymnus continentalis* Fruhstorfer, 1912 (Figure 8e), and *Ticherra acte acte* (Moore, [1858]) (Figure 8f) further enrich the region's butterfly diversity. Swallowtails, including *Graphium* Scopoli, 1777 spp., *Papilio* Linnaeus, 1758 spp. (Figure 8g,h), and pierids such as *Delias descombesi descombesi* (Boisduval, 1836) (Figure 8i), are frequently observed, making the area a year‐round butterfly hotspot. The region's diverse species also lend themselves to the creation of butterfly gardens with native larval host plants and nectar sources, which could be incorporated into local cafes, hotels, and parks. These gardens would attract visitors while providing an immersive experience, promoting both tourism and conservation.

This ecotourism initiative could be effectively combined with other wildlife tourism ventures, given the region's rich biodiversity (Giri and Chalise 2008; Basaula et al. 2021). The continuous discovery of new butterfly species (KC and Pariyar 2019; KC 2020, 2021, 2022, 2023) suggests that the Methlang Forest area remains underexplored. The development of butterfly tourism could not only boost local conservation efforts but also encourage citizen science, particularly through platforms such as iNaturalist. Such initiatives could contribute to the growing database on local butterfly diversity, providing crucial data for the design of effective conservation strategies, especially as the area faces increasing pressures from urbanization and habitat fragmentation.

While a closed butterfly park featuring exotic species is one possible model, this study recommends an open park that showcases native species and natural vegetation, as it would be more cost‐effective and environmentally sustainable. This approach has been successfully implemented in butterfly parks around the world, such as the National Butterfly Center in Texas, USA (KC and Sapkota 2024b), and parks in India such as Ramsai Butterfly Park in the Dooars, Ovalekar Butterfly Farm in Wadi, Thane, and Saharanpur Butterfly Park in Uttar Pradesh (Kanaujia et al. 2022). These parks not only promote environmental education but also serve as valuable community resources. Integrating butterfly parks into school programs would help foster a deeper connection to nature among young learners, encouraging environmental stewardship and lifelong appreciation for biodiversity. By adopting such a model, Methlang Forest could establish itself as a premier destination for butterfly tourism, promoting local economic development while ensuring the long‐term conservation of this ecologically sensitive habitat—a concept also explored by KC and Sapkota (2024a) in their study area.

## 5 Conclusions

This study, documenting 225 butterfly species in the Methlang Forest area, offers valuable baseline data for future studies, particularly considering urbanization and habitat fragmentation. The seasonal peaks in butterfly abundance coincide with periods that could attract ecotourists, particularly during spring and fall when the forest is most vibrant. With its rich biodiversity, the forest presents significant potential for butterfly‐focused ecotourism, which could extend across the broader Himalayan region, raising awareness about conservation and encouraging sustainable tourism practices. These baseline data are crucial for monitoring long‐term ecological changes and ensuring the forest remains a viable, attractive destination for ecotourism, supporting both conservation efforts and local communities. Additionally, the ongoing discoveries of new butterfly species in the study area further emphasize the importance of its conservation, showcasing the area's biodiversity potential and the need for continuous protection and study.

## Author Contributions

**Sajan KC:** conceptualization (lead), data curation (lead), formal analysis (lead), investigation (lead), methodology (lead), software (lead), validation (lead), visualization (lead), writing – original draft (lead), writing – review and editing (lead).

## Conflicts of Interest

The author declares no conflicts of interest.

## Data Availability

Data are archived in Figshare in draft status under doi: 10.6084/m9.figshare.28139078, and can be accessed using the following link: https://figshare.com/s/d3535d94cbfd465add0c.
